# Long-term outcomes of active surveillance for clinically localized prostate cancer in a community-based setting: results from a prospective non-interventional study

**DOI:** 10.1007/s00345-020-03471-x

**Published:** 2020-09-30

**Authors:** Jan Herden, Andreas Schwarte, Thorsten Werner, Uwe Behrendt, Axel Heidenreich, Lothar Weissbach

**Affiliations:** 1 University of Cologne, Faculty of Medicine and University Hospital Cologne, Department of Urology, Uro-Oncology, Robot-Assisted and Reconstructive Urology, Cologne, Germany; 2 PAN Clinic, Urological Practice, Cologne, Germany; 3 Urological Practice Borken, Borken, Germany; 4 Department of Urology, St. Agnes Hospital Bocholt, Bocholt, Germany; 5 Urological Practice Herzberg, Herzberg Am Harz, Germany; 6 Urological Practice Wittenbergplatz, Berlin, Germany; 7 Health Research for Men GmbH, Berlin, Germany

**Keywords:** HAROW study, Active surveillance, Conservative management, Health service research, Outcomes research, Routine care

## Abstract

**Purpose:**

To report on long-term outcomes of patients treated with active surveillance (AS) for localized prostate cancer (PCa) in the daily routine setting.

**Methods:**

HAROW (2008–2013) was a non-interventional, health service research study about the management of localized PCa in the community setting, with 86% of the study centers being office-based urologists. A follow-up examination of all patients who opted for AS as primary treatment was carried out. Overall, cancer-specific, and metastasis-free survival, as well as discontinuation rates, were determined.

**Results:**

Of 329 patients, 62.9% had very-low- and 21.3% low-risk tumours. The median follow-up was 7.7 years (IQR 4.7–9.1). Twenty-eight patients (8.5%) died unrelated to PCa, of whom 19 were under AS or watchful waiting (WW). Additionally, seven patients (2.1%) developed metastasis. The estimated 10-year overall and metastasis-free survival was 86% (95% CI 81.7–90.3) and 97% (95% CI 94.6–99.3), respectively. One hundred eighty-seven patients (56.8%) discontinued AS changing to invasive treatment: 104 radical prostatectomies (RP), 55 radiotherapies (RT), and 28 hormonal treatments (HT). Another 50 patients switched to WW. Finally, 37.4% remained alive without invasive therapy (22.2% AS and 15.2% WW). Intervention-free survival differed between the risk groups: 47.8% in the very-low-, 33.8% in the low- and 34.6% in the intermediate-/high-risk-group (*p* = 0.008). On multivariable analysis, PSA-density ≥ 0.2 ng/ml^2^ was significantly predictive for receiving invasive treatment (HR 2.55; *p* = 0.001).

**Conclusion:**

Even in routine care, AS can be considered a safe treatment option. Our results might encourage office-based urologists regarding the implementation of AS and to counteract possible concerns against this treatment option.

**Electronic supplementary material:**

The online version of this article (10.1007/s00345-020-03471-x) contains supplementary material, which is available to authorized users.

## Introduction

Active surveillance (AS) is a non-invasive treatment strategy for patients with well-differentiated, localized prostate cancer (PCa). In contrast to watchful waiting (WW) being a palliative option for patients with reduced life expectancy, AS implies curative intention. Prostate-specific antigen (PSA) assessment, digital rectal examination (DRE), and re-biopsies are performed frequently to switch to invasive treatment when signs of progression occur [1, 2].

Prospective clinical long-term AS studies have confirmed a 10-year cancer-specific-survival of > 98%, which is comparable to that of an immediate invasive treatment [3–8].

Noteworthy, most of these studies are clinical trials from large academic centers with stringent inclusion and exclusion criteria. Since in “real life” AS is mainly applied by office-based urologists, the question arises whether this leads to similarly promising results in daily routine care.

The HAROW study (2008–2013) is a prospective, multicenter, health service research study with the aim of investigating the treatment of localized PCa in the community setting in Germany [9]. The name represents the five possible treatment options: hormonal treatment (HT), AS, radiotherapy (RT), radical prostatectomy (OP = RP), and WW. Because it was conceived as a non-interventional observational study, no specifications were given regarding the choice of treatment or how it was carried out. The AS group was of particular interest because at the time of recruitment this strategy was relatively unknown and not widely used [10].

We herein report on the long-term outcomes of the AS cohort with up to 11 years of follow-up, including survival and metastatic outcomes, as well as discontinuation rates and risk factors for deferred invasive treatment.

## Patients and methods

### HAROW study

From July 2008-July 2013 patients with newly diagnosed localized (≤ T2c) PCa were prospectively enrolled by 259 study centers, 86% of which were office-based urologists. Half of them (*n* = 131) recruited patients in AS. Although at that time AS was already considered in the guidelines of the European Association of Urology (EAU) [11], it was still a new and largely non-accepted treatment strategy among German urologists. Because of the non-interventional character of the study, only recommendations regarding inclusion, follow-up, and discontinuation of AS were given corresponding to those available in the literature [12] and the European PRIAS study (Prostate Cancer Research International Active Surveillance), the then-largest published prospective trial of AS [13]. Inclusion criteria for AS were T-category ≤ cT2c, PSA ≤ 10 ng/ml, Gleason grade group 1, PSA-density ≤ 0.2 ng/ml^2^ and ≤ 2 positive biopsies. The recommended follow-up procedure included DRE, PSA, and PSA doubling time (PSA-DT) every 3 months in the first 2 years, and every 6 months thereafter. Re-biopsy was recommended after 1 year, and then every 3 years. In case of histological evidence of progressive disease, increasing PSA levels with PSA-DT < 3 years, or clinical signs of progression on DRE discontinuation of AS was recommended, alternatively on patient’s request. Multiparametric magnetic resonance imaging (mpMRI) of the prostate was neither used nor recommended as it was not available at the time of recruitment. Data of recruitment, diagnostics, and course of disease in the total cohort for the study period with a median observation period of 28.4 months have been published elsewhere [9, 10, 14].

### Follow-up of the AS group

A follow-up survey of all AS patients including those who had switched to another form of treatment was carried out until August 2019. Questionnaires were sent to the patients by mail. All non-responders were contacted again and interviewed by telephone. In the case of missing response or lacking information on the course of the disease including the cause of death, treating study physicians were contacted. The following parameters were collected: Overall, cancer-specific, metastasis-free, and intervention-free survival, reasons for discontinuation of AS, and type of deferred treatment.

### Statistical analysis

Data were analyzed using IBM’s statistical program SPSS, version 22. Metric variables were evaluated by means of univariate ANOVA, and categorical variables analyzed using the chi-squared test or Fisher’s-exact test. Kaplan–Meier method and log-rank test were used to analyze overall, metastasis-free, and intervention-free survival. We used logistic regression as a multivariate analysis to determine independent factors influencing the target variable “receiving interventional treatment”. The significance level was set at 5% for all calculations.

## Results

**Fig. 1:**
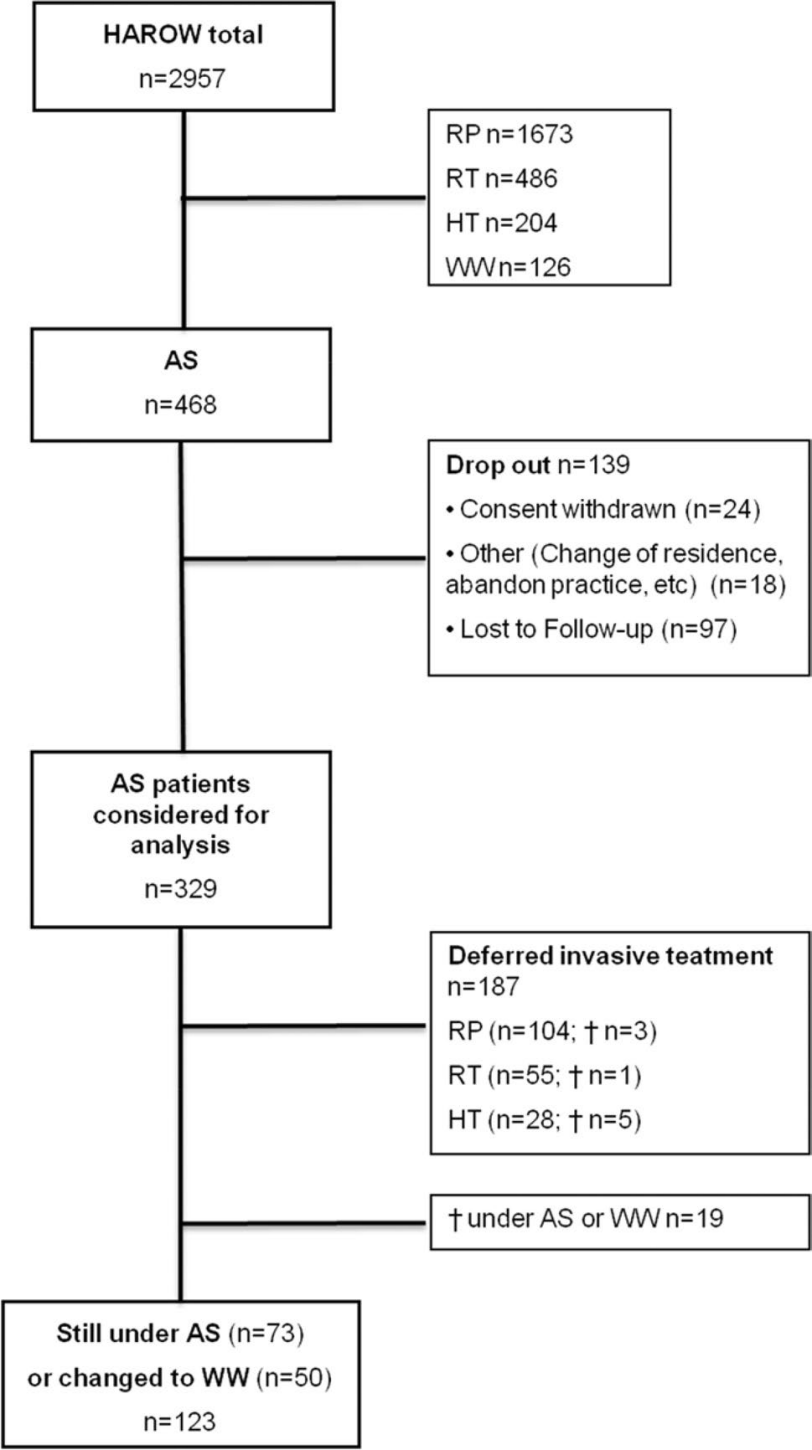
Flow chart of the HAROW study and outcome of the active surveillance (AS) patients at the time of the last follow-up. *RP* radical prostatectomy, *RT* radiotherapy, *HT* hormone treatment, *WW* watchful waiting. ^†^Death

Of 2957 patients enrolled, 468 (15.8%) chose AS. During the course of the HAROW-study and follow-up period reasons for drop-out included: consent withdrawn (5.1%), lost to follow-up (20.7%), and other reasons, e.g. change of residence, physician abandoned the practice, etc. (3.8%). Finally, data from 329 patients were available for evaluation (Fig. 1).

Patient characteristics at baseline are presented in Table 1. Two hundred and seven (62.9%) patients accounted for the very-low-, 70 (21.3%) for the low-, and 52 (15.8%) for the intermediate- or high-risk- group, respectively, according to the National Comprehensive Cancer Network (NCCN) guidelines [2].

**Table 1:** Patient characteristics at baseline

	Total (*n* = 329)		Patients remaining on AS (*n* = 92)		Patients changing to WW (*n* = 50)		Patients changing to invasive treatment (*n* = 187)		*p* value
	Median	(IQR)	Median	(IQR)	Median	(IQR)	Median	(IQR)	
Age (years)	69.0	(63.4–72.5)	67.9	(61.6–72.1)	71.9	(67.7–75.1)	68.3	(63.3–71.9)	0.010
PSA (ng/ml)	5.3	(3.9–7.2)	5.2	(2.7–7.0)	4.6	(2.5–6.4)	5.6	(4.5–7.5)	0.001
Follow up (months)	91.8	(55.9–109.3)	99.8	(80.2–110.0)	92.0	(69.5–111.4)	86.5	(47.6–109.3)	0.19
	*n*	(%)	*n*	(%)	*n*	(%)	*n*	(%)	
Tumor category									0.013
≤ cT1c	277	(84.2)	81	(88.1)	44	(88.0)	152	(81.3)	
cT2a	36	(11.0)	8	(8.7)	4	(8.0)	24	(12.8)	
cT2b	9	(2.7)	2	(2.2)	2	(4.0)	5	(2.7)	
cT2c	7	(2.1)	1	(1.1)	0	(0)	6	(3.2)	
Gleason grade group									0.890
1	307	(93.3)	87	(94.5)	47	(94.0)	174	(93.0)	
2	21	(6,4)	5	(5.5)	3	(6.0)	13	(7.0)	
Number of positive cores per biopsy									< 0.001
0*	38	(11.6)	16	(17.4)	14	(28.0)	8	(4.3)	
1	186	(56.5)	43	(46.7)	26	(52.0)	117	(62.6)	
2	84	(25.5)	25	(27.2)	6	(12.0)	53	(28.3)	
≥ 3	15	(4.6)	4	(4.3)	2	(4.0)	9	(4.8)	
n.a	6	(1.8)	4	(4.3)	2	(4.0)	0	(0)	
PSA (ng/ml)									0.431
≤ 10	308	(93.6)	89	(96.7)	46	(92.0)	173	(92.5)	
10–20	18	(5.5)	3	(3.3)	4	(8.0)	11	(5.9)	
> 20	3	(0.9)	0	(0)	0	(0)	3	(1.6)	
PSA-density (ng/ml^2^)									0.005
< 0.2	233	(70.8)	69	(75.0)	43	(86.0)	121	(64.7)	
≥ 0.2	71	(21.6)	16	(17.4)	4	(8.0)	51	(27.3)	
n.a	25	(7.6)	7	(7.6)	3	(6.0)	15	(8.0)	
CCI									0.158
0	256	(77.8)	75	(81.5)	37	(74.0)	144	(77.0)	
1	52	(15.8)	9	(9.8)	8	(16.0)	35	(18.7)	
≥ 2	17	(5.2)	7	(7.6)	4	(8.0)	6	(3.2)	
n.a	4	(1.2)	1	(1.1)	1	(2.0)	2	(1.1)	
Risk group									0.134
Very low	207	(62.9)	63	(68.5)	36	(72.0)	108	(57.8)	
Low	70	(21.3)	19	(20.7)	6	(12.0)	45	(24.1)	
Intermediate/high	52	(15.8)	10	(10.9)	8	(16.0)	34	(18.2)	

*PSA* prostate-specific antigen, *n.a.* not available

*Patients with no positive biopsies were diagnosed as incidental prostate cancer diagnosed by transurethral resection of the prostate

**Fig. 2:**
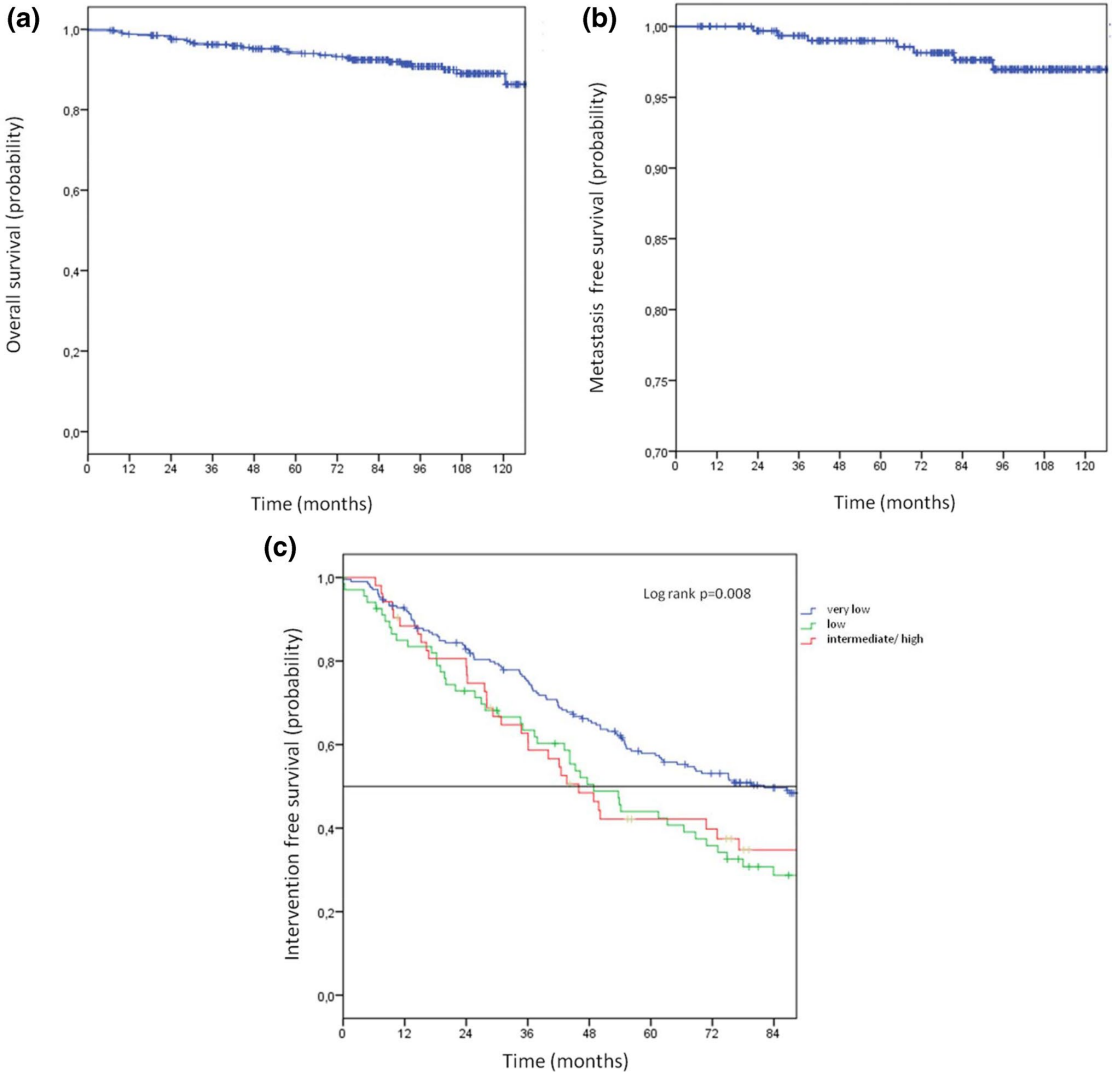
Kaplan–Meier curves illustrating the **a** overall survival, **b** metastasis-free survival, and **c** the intervention-free survival stratified by risk groups for 329 active surveillance patients

Median follow-up was 7.7 years (interquartile-range = IQR 4.7–9.1, Min–Max 0.1–11.0). In this period, 28 patients (8.5%) died at a median age of 74 years (IQR 72–78) after a median follow-up of 4.1 years (IQR 2.3–6.3), of which 19 were still under AS or WW. No PCa-specific cause of death could be detected. The main causes of death were other malignancies (36%) and cardiovascular diseases (25%) (Supplementary Table 1). Seven patients (2.1%) developed metastasis after a median of 5.4 years (IQR 2.4–6.8), including five with very-low and two with intermediate-risk tumors (Supplementary Table 2). The Kaplan–Meier estimated 10-year overall and metastasis-free survival was 86% (95% CI 81.7–90.3) and 97% (95% CI 94.6–99.3), respectively (Fig. 2a + b).

A total of 187 patients (56.8%) discontinued AS in favor of invasive treatment: 104 chose RP, 55 RT, and 28 HT. The main reasons for discontinuation were biopsy-upgrade and PSA-elevation in the RP-group (40.4% and 33.7%) and the RT-group (29.1% each), and PSA-elevation and physician´s advice (35.7% and 19.7%) in the HT-group (Supplementary Fig. 1). Additionally, 50 patients switched from AS to WW and maintained a non-invasive approach. These patients were older and had a lower baseline PSA and PSA-density compared to patients who remained on AS or switched to invasive treatment. No differences were seen regarding comorbidities (CCI) at baseline or among the distribution to the risk-groups (Table 1).

The median time to change to RP (33.0 months) and RT (38.5 months) was significantly shorter than to HT (59.1 months) and WW (70.1 months, *p* < 0.001).

Within the study period (2008–2013, mFU 28.5 months) information about follow-up examinations (re-biopsy and repeat PSA tests) as well as histology after deferred RP was stated: 71% had ≥ 4 PSA measurements and 55% had at least one re-biopsy. Histological results from RP specimen were available from 65 patients. Locally advanced disease (≥ pT3) was seen in 8/65 (12.3%) and Gleason grade group ≥ 3 in 13/65 (20.0%), with no significant differences among the risk-groups (*p* = 0.6 and *p* = 0.22; Table 2).

**Table 2:** Pathological findings in deferred radical prostatectomy after the termination of active surveillance for all three risk groups (*n* = 65)

	Total (*n* = 65)	Very low risk (*n* = 37)	Low risk (*n* = 18)	Int./high risk (*n* = 10)	*p* value
T-Category					0.60
pT2a	5	3	2	0	
pT2b	4	0	2	2	
pT2c	37	24	10	3	
≥ pT3	8	6	2	0	
n.a	11	4	2	5	
Gleason grade group					0.22
1	19	11	7	1	
2	24	14	6	4	
3	10	8	1	1	
≥ 4	3	2	1	0	
n.a	9	2	3	4	

*n.a.* not available

At the end of the observation period, 123 patients (37.4%) were alive without an invasive therapy, including 73 (22.2%) AS- and 50 (15.2%) WW-patients (Fig. 1). However, intervention-free-survival differed between the risk groups: 47.8% for the very-low—, 33.8% for the low—and 34.6% for the intermediate-/high-risk group (p = 0.008) (Fig. 2c). In multivariable analysis, only PSA-density ≥ 0.2 ng/ml^2^ was significantly predictive for receiving an invasive treatment (HR 2.55; *p* = 0.001) (Table 3).

**Table 3:** Multivariate analysis for the association between baseline characteristics and deferred invasive treatment

Category	Adjusted HR	95% CI	*p* value
Age			
Continuos	1.0	0.99–1.01	0.949
Charlson comorbidity index			
0	Reference		
1	1.78	0.98–3.21	0.055
≥ 2	0.39	0.14–1.10	0.073
PSA			
≤ 10 ng/ml	Reference		
> 10 ng/ml	0.92	0.23–3.70	0.9
PSA density			
< 0.2 ng/ml^2^	Reference		
≥ 0.2 ng/ml^2^	2.55	1.49–4.36	0.001
Gleason grade group			
1	Reference		
2	0.87	0.18–4.31	0.871
cT-category			
cT1	Reference		
cT2	1.21	0.59–2.46	0.607
Risk category			
Very low	Reference		
Low	0.56	0.16–1.91	0.356
Interm./high	1.05	0.44–2.50	0.916
Number of positive biopsies			
≤ 1	Reference		
2	1.37	0.85–2.21	0.2
≥ 3	1.01	0.31–3.28	0.984

*HR* hazard ratio, *CI* confidence interval, *PSA* prostate-specific antigen

## Discussion

In the HAROW-study we evaluated the outcomes of patients with localized PCa under AS in the routine-care setting. A distinctiveness of the study is the design as a health-service-research study. Other than most single-center AS-studies from academic or tertiary-care centers, HAROW aims to represent a “real-world”-situation with the inclusion of mainly office-based urologists across Germany. This is intended to increase the generalizability of the results since in routine-care office-based urologists in particular are at the forefront in managing AS. In addition to diagnosis and advice on management options of localized PCa, they are responsible for an essential part of the treatment and follow-up of AS-patients.

Short term outcomes of the AS cohort within the study-period (2008–2013, mFU 28.5 months) have already been published. It could be demonstrated that AS recommendations were largely respected. AS-patients who were contrarily admitted to these recommendations included 12.8% with intermediate- and 3% with high-risk tumors, only 2.1% had a PSA > 20 ng/ml, and no Gleason-grade-group 3 were assigned to AS [10].

We now present the long-term outcomes which confirm AS as a safe treatment option also in the routine-care, since no PCa-specific death was observed and only 2.1% of the patients developed metastasis. On the other hand, our results indicate that with time, only 22% adhered to AS, whereas additionally 15% switched from AS to WW and remained without invasive treatment.

With a median follow-up of 7.7 years, our cohort constitutes one of the very few studies that so far have reported intermediate to long-term outcomes of men managed with AS. A list of prospective cohorts reporting on AS outcomes is shown in Supplementary Table 3, demonstrating that nearly half of these are single-center studies from tertiary care centers [3, 4, 7, 15–20]. The two most important single-center studies are from Sunnybrook Toronto and from Johns-Hopkins University, reporting on 15-year actuarial rates for cancer-specific survival of 94% [3] and 99.9% [7], respectively.

Of all multicenter-studies, the PRIAS-study is the one that probably best represents the real-life situation and is therefore closest to our study design [17]. This international AS study began in 2006 and included more than 100 centers in 17 countries worldwide with inclusions from academic, nonacademic, and private practices. ProtecT is the only study that compared RP, RT, and active monitoring, an adapted form of AS, in a prospective randomized setting with a 10-year follow-up. A significant difference between the treatment groups regarding survival rates was not found. However, disease progression and metastases occurred less frequently in patients who received RP or RT [4].

Some of our findings were noteworthy. First, regarding overall, cancer-specific, and metastasis-free survival our results are in line with all other AS-series revealing the same promising results. However, the cancer-specific survival in our study is 100% and therefore slightly higher compared to most other AS-series with medium- to long-term follow-up, reporting 1–2% PCa death. Thus, it could be assumed that some PCa-specific deaths may not have been identified. To scrutinize this point, all treating urologists of the deceased patients were contacted. In 19/28 patients' causes of death could be determined, revealing no PCa-specific deaths. Furthermore, in none of the remaining nine patients an event of metastasis has been reported, so that even in these patients death of PCa seems to be unlikely (Supplementary Table 1).

Second, our intervention-free-survival of 37.4%, including 22.2% under AS and 15.2% under WW, was lower compared to most other series, revealing intervention-free survival rates between 47 and 63% after 10 years. Interestingly, our results are close to that of the PRIAS-study, where 27% of men adhered to AS, and 15% switched to WW (or died of another cause) after 10 years of follow-up. In this regard, Hemelrijck et al. recently reported about discontinuation rates from a worldwide AS-database including 21 Centers in 12 countries, in which 39% of the patients were still on AS or WW after 10 years [21].

The observation of increasing discontinuation rates with an increasing number of study-centers may be an indication that switching to invasive therapy is more common in the routine care setting than in clinical studies from academic-centers. One reason could be that patients and physicians outside the academic setting feel less confident in dealing with AS. This becomes evident when examining the reasons for discontinuation: in HAROW 16.6% discontinued without biopsy or PSA-progression due to patient wish or physician´s advice. Likewise, in PRIAS 5% discontinued due to anxiety or patient request, and 12% discontinued for other reasons without having a re-classification [17]. In contrast, in the Toronto-series only 6% discontinued upon patient preference without signs of progression [3].

This uncertainty, primarily affecting patients with higher risk features is reflected in our observation that low- and intermediate/high-risk patients discontinued AS much earlier than very low-risk patients (Fig. 2c). In this context, we were able to demonstrate in a previous investigation of our cohort, that the main reason for intervention in the intermediate-/high-risk group was patient preference without sings of progression [22].

Third, we demonstrated that the preference for invasive deferred treatment decreased with time. This indicates that in older age also HT and WW become part of AS. For a patient who initially selected AS and is no longer a candidate for invasive therapy due to increasing age or emerging comorbidities, the transition from AS to WW becomes an obvious option. On the basis of the Swedish national healthcare register, van Hemelrijk et al. estimated that 48% of men that started AS with a very-low-risk PCa change to WW after a median of eight years [23]. In this context, we could demonstrate in our cohort that patients switching to WW were significantly older than patients who remained in AS or opted for invasive therapy (Table 1).

Fourth, although re-biopsies and PSA measurements were only determined within the time of recruitment (2008–2013), it could be shown that follow-up examinations were fewer than expected: only 55% received at least one re-biopsy, and 71% received ≥ 4 repeat PSA tests within this period. Similar observations of less intense follow-up outside controlled clinical trials could be demonstrated by Loeb et al. on the basis of a Surveillance, Epidemiology, and End Results (SEER)-Medicare database analysis. Among 5192 AS patients > 80% had more than one PSA test per year but < 13% received biopsy beyond the first 2 years [24]. However, at least in our cohort, the reduced follow-up studies do not seem to have any impact on the oncologic outcome.

Finally, on the multivariable analysis we could identify PSA-density ≥ 0.2 ng/ml^2^ as a predictor for receiving invasive treatment. This confirms the results of other AS-series, in which PSA-density was positively associated with the risk of biopsy re-classification [7, 19, 25].

The strength of our study includes its prospective nature, its non-interventional design, the long follow-up period, and the high number of study-centers, consisting mainly of office-based urologists, thus reflecting the reality of everyday conditions better than results of prospective AS studies from single tertiary-care centers.

One limitation of our study is the relatively high drop-out rate of 29.7%. Considering this rate more closely, it becomes evident that in nearly one-third of these cases the reasons for drop-out could be stated and only 20% were lost to follow-up, which is in line with other health care studies [26]. Noteworthy, in PRIAS only 107 of 5302 patients were followed for more than 7.5 years and even the prospective randomized ProtecT-trial report about 14% lost to follow-up [4, 17]. Further limitations include the lacking information about histologic results after RP or re-biopsy, as well as the frequency of follow-up examinations beyond the study period of 2008–2013. It should also be noted that our study was conducted in the era before mpMRI became available as diagnostic tools which since have shown promising results in better patient selection and monitoring for men who undergo AS [27]. Today, the use of mpMRI at baseline and for the assessment during surveillance is recommended by most guidelines [1, 2], since the incorporation of mpMRI appears to be cost-effective, improves patient selection, and may reduce the necessity biopsies in the course of follow-up [28–30].

## Conclusion

Our results demonstrate that AS is a safe treatment option for localized PCa within the real-life healthcare situation, outside of controlled trials which investigate pre-selected study participants and a set treatment protocol. We hope that this might encourage office-based urologists in particular regarding the implementation of AS and to counteract possible concerns against this treatment option. On the other hand, our results indicate that discontinuation rates are higher in the routine-care setting and more likely due to reasons other than re-classification on biopsy or PSA rise. In the future, it will be necessary to identify reasons for the prevailing uncertainty in dealing with AS and to further improve diagnostic tools to allow more patients without a progressive disease to pursue a non-invasive approach.

## Electronic supplementary material

Below is the link to the electronic supplementary material.

Supplementary file1 Supplementary Fig. 1 Main reasons for an invasive treatment for 187 patients that discontinued active surveillance and (b) time to change to deferred treatment with additional 50 patients that switched to watchful waiting (RP=radical prostatectomy, RT=radiotherapy, HT=hormone treatment, WW=watchful waiting, n.a.=not available) (DOCX 45 kb)

Supplementary file2 (DOCX 16 kb)

Supplementary file3 (DOCX 13 kb)

Supplementary file4 (DOCX 13 kb)
