# Supplementary figures and images for: Long-term outcomes of active surveillance for clinically localized prostate cancer in a community-based setting: results from a prospective non-interventional study
Source: World J Urol. 2020 Sep 30;39(7):2515–23. doi: 10.1007/s00345-020-03471-x (PMC8332563; doi:10.1007/s00345-020-03471-x)

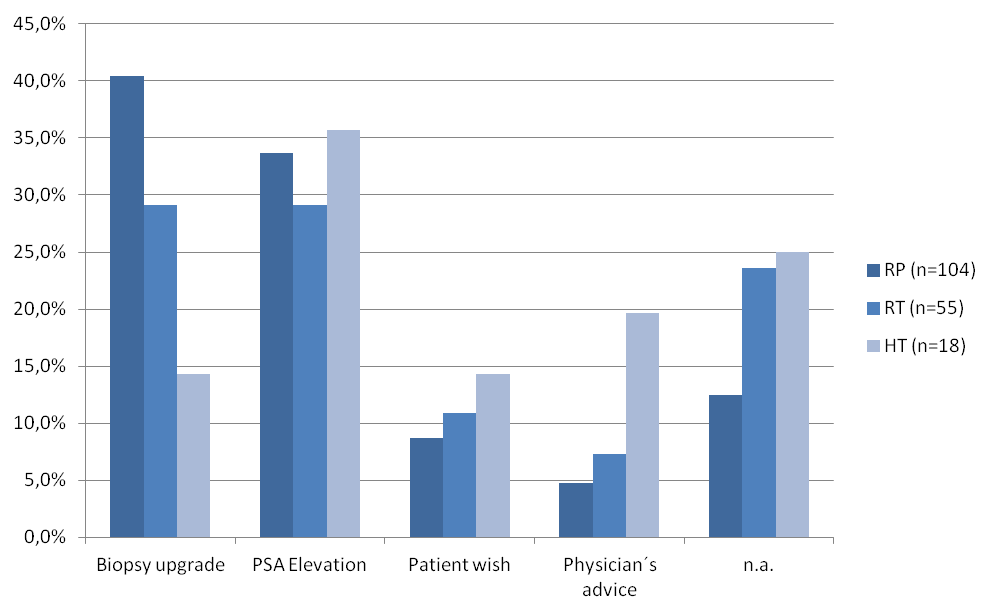

Supplement: Supplementary file 1 — Supplementary file1 Supplementary Fig. 1 Main reasons for an invasive treatment for 187 patients that discontinued active surveillance and (b) time to change to deferred treatment with additional 50 patients that switched to watchful waiting (RP=radical prostatectomy, RT=radiotherapy, HT=hormone treatment, WW=watchful waiting, n.a.=not available) (DOCX 45 kb) [file 345_2020_3471_MOESM1_ESM.docx]
