# Supplementary material for: Long-term outcomes of active surveillance for clinically localized prostate cancer in a community-based setting: results from a prospective non-interventional study
Source: World J Urol. 2020 Sep 30;39(7):2515–23. doi: 10.1007/s00345-020-03471-x (PMC8332563; doi:10.1007/s00345-020-03471-x)
Supplement: Supplementary file 2 — Supplementary file2 (DOCX 16 kb) [file 345_2020_3471_MOESM2_ESM.docx]

**Supplementary Tab. 1** Case histories of 28 patients that deceased during the course of follow-up

| Patient | Year | Age upon | Time from PCa | Risk | CCI | Cause of death | Metastasis | Last PCa | Commentary |
| --- | --- | --- | --- | --- | --- | --- | --- | --- | --- |
| Nr. | of death | death (years) | diagnosis to death (years) | category | (study entry) |  | due to PCa ? | treatment |  |
|  |  |  |  |  |  |  |  |  |  |
| 1 | 2015 | 72 | 5.5 | very low | 1 | cancer of unknown primacy | no | AS | PSA two month prior to death: 0.64 ng/ml |
| 2 | 2017 | 80 | 8.9 | very low | 0 | cardiac arrest | no | HT | PSA four month prior to death: 0.32 ng/ml |
| 3 | 2011 | 69 | 2.5 | low | 0 | leukaemia | no | AS |  |
| 4 | 2011 | 75 | 2.6 | very low | 1 | unknown* | no | AS |  |
| 5 | 2010 | 69 | 0.7 | very low | 0 | myocardial infarction | no | AS |  |
| 6 | 2017 | 85 | 8.5 | very low | 1 | cardiac arrest | no | HT | PSA one month prior to death: 0.32 ng/ml |
| 7 | 2016 | 62 | 7.6 | very low | 2 | diabetes mellitus | no | AS |  |
| 8 | 2019 | 85 | 10.0 | high | 0 | renal failure | no | HT | "high risk" PCa due to T-Kategory "cT2c" |
| 9 | 2015 | 80 | 6.3 | intermediate | 0 | unknown* | no | AS |  |
| 10 | 2017 | 59 | 7.3 | low | 0 | brain tumor | no | AS |  |
| 11 | 2013 | 75 | 3.8 | very low | 1 | lymphoma | no | AS |  |
| 12 | 2013 | 78 | 3.4 | low | 1 | unknown* | no | AS |  |
| 13 | 2012 | 78 | 2.4 | intermediate | 0 | unknown* | no | HT |  |
| 14 | 2013 | 56 | 1.8 | very low | 0 | unknown* | no | AS |  |
| 15 | 2018 | 74 | 7.8 | very low | 0 | leukaemia | no | RP | PSA three month prior to death: 0.01 ng/ml |
| 16 | 2015 | 79 | 4.8 | intermediate | 3 | bladder cancer | no | AS |  |
| 17 | 2017 | 82 | 6.3 | very low | 1 | unknown* | no | AS |  |
| 18 | 2013 | 60 | 2.0 | low | 0 | unknown* | no | AS |  |
| 19 | 2016 | 77 | 4.7 | intermediate | 3 | cardiac insufficiency | no | WW |  |
| 20 | 2013 | 77 | 2.0 | very low | 0 | lung cancer | no | AS |  |
| 21 | 2016 | 74 | 3.7 | very low | 0 | soft tissue sarkoma | no | HT | PSA two month prior to death: 0.14 ng/ml |
| 22 | 2012 | 72 | 1.2 | very low | 0 | cardiac arrest | no | RP | PSA three month prior to death: 0.05 ng/ml |
| 23 | 2013 | 72 | 0.8 | very low | 0 | lung cancer | no | AS |  |
| 24 | 2012 | 72 | 4.4 | low | 0 | lung cancer | no | AS |  |
| 25 | 2017 | 78 | 4.9 | low | 3 | unknown* | no | RP |  |
| 26 | 2013 | 72 | 0.7 | intermediate | 2 | myocardial infarction | no | AS |  |
| 27 | 2018 | 71 | 5.9 | very low | 0 | myocardial infarction | no | RT |  |
| 28 | 2015 | 72 | 2.4 | intermediate | 1 | unknown* | no | RP |  |

PCa = prostate cancer, CCI = Charlson Comorbidity Index, PSA = prostate specific antigen, AS = active surveillance, WW = watchful waiting, HT = hormone treatment, RT = radiotherapy, RP = radical prostatectomy

* in patients whose cause of death was unknown, cause of death from known prostate cancer could be excluded
