# Supplementary material for: Long-term outcomes of active surveillance for clinically localized prostate cancer in a community-based setting: results from a prospective non-interventional study
Source: World J Urol. 2020 Sep 30;39(7):2515–23. doi: 10.1007/s00345-020-03471-x (PMC8332563; doi:10.1007/s00345-020-03471-x)
Supplement: Supplementary file 3 — Supplementary file3 (DOCX 13 kb) [file 345_2020_3471_MOESM3_ESM.docx]

**Supplementary Tab. 2** Case histories of seven patients that developed metastasis under active surveillance

| Patient | Age at PCa diagnosis | CCI | PSA at PCa diagnosis | Prostate- | PSA-density | Tumor- | Gleason- | Number of positive | Risk | Deferred PCa |
| --- | --- | --- | --- | --- | --- | --- | --- | --- | --- | --- |
| No. | (years) |  | (ng/mL) | volume (mL) | (ng/mL/mL) | Category | grade group | cores per biopsy | group | treatment |
|  |  |  |  |  |  |  |  |  |  |  |
| 1 | 69 | 0 | 8.8 | 20 | 0.44 | cT2a | 2 | 1 | intermediate | HT |
| 2 | 87 | 0 | 12.9 | 89 | 0.14 | cT2a | 1 | 1 | intermediate | HT |
| 3 | 71 | 0 | 2.4 | n.a. | n.a. | cT1a | 1 | 0 | very low | HT |
| 4 | 58 | 0 | 6.1 | 40 | 0.15 | cT1c | 1 | 1 | very low | RT |
| 5 | 66 | 0 | 4.2 | 31 | 0.14 | cT1c | 1 | 1 | very low | RP |
| 6 | 65 | 0 | 5.2 | 36 | 0.14 | cT2a | 1 | 1 | very low | RP |
| 7 | 71 | 0 | 8.2 | 43 | 0.19 | cT1c | 1 | 1 | very low | RP |

PCa = prostate cancer, CCI = Charlson Comorbidity Index, PSA = prostate specific antigen, n.a. = not available, HT = hormone treatment, RT = radiotherapy, RP = radical prostatectomy
