# Supplementary material for: Long-term outcomes of active surveillance for clinically localized prostate cancer in a community-based setting: results from a prospective non-interventional study
Source: World J Urol. 2020 Sep 30;39(7):2515–23. doi: 10.1007/s00345-020-03471-x (PMC8332563; doi:10.1007/s00345-020-03471-x)
Supplement: Supplementary file 4 — Supplementary file4 (DOCX 13 kb) [file 345_2020_3471_MOESM4_ESM.docx]

**Supplementary Tab. 3** Prospective cohorts reporting on active surveillance outcomes

| **Cohort** | **n** | **Median Follow-up (years)** | **Overall survival** | **Cancer specific survival** | **Metastasis free survival** | **Intervention free survival** | **single (s)- or multi-**  **center (m)** |
| --- | --- | --- | --- | --- | --- | --- | --- |
|  |  |  |  |  |  |  |  |
| Sunnybrook Toronto [3] | 993 | 6.4 | 10y: 80%  15y: 62% | 10y: 98.1%  15y: 94.3% | 98.7% | 5y: 75.7%  10y: 63.5%  15y: 55% | s |
| Johns Hopkins University [7] | 1818 | 5.0 | 10y: 93.2%  15y: 72% | 10y+15y: 99.9% | 10y+15y: 99.4% | 5y: 59%  10y: 52%  15y: 48% | s |
| University of Miami [15] | 230 | 3.7 | n.a. | 100% | n.a. | 5y: 86% | s |
| Royal Mardsen [16] | 471 | 5.7 | 8y: 91% | 8y: 98% | n.a. | 5y: 70% | s |
| ProtecT [4] | 545 | 10.0 | 10y: 89% | 10y: 99% | 10y: 94% | 10y: 47% | m |
| PRIAS [17] | 5302 | 622 followed on AS >5 yr  107 followed for >7.5 yr | 5y: 97%  10y: 89% | 5y: 99%  10y: 99% | n.a. | 5y: 48 %  10y: 27 % | m |
| Gothenburg [18] | 474 | 8.0 | 10y: 80%  15y: 51% | 10y: 99.5%  15y: 96% | 10y: 99 %  15y: 93% | 10y: 47 %  15y: 34% | m |
| PASS [19] | 905 | 2.3 | 99% | 100% | 100% | 2y: 88%  5y: 71 %  10y: 50% | m |
| Denmark [20] | 936 | 7.5 | n.a. | 10y: 99% | n.a. | 10y: 63% | m |
| HAROW [present study] | 329 | 7.7 | 10y: 89% | 100% | 10y: 97% | 37.4% | m |

n.a. = not available
